# Exploring English for academic purposes learners’ engagement with ChatGPT-4 in academic writing revision: a case study from a private language institute in China

**DOI:** 10.3389/frai.2026.1802007

**Published:** 2026-06-01

**Authors:** Jinming Lawrence Du, Yiran Chen

**Affiliations:** 1 University of Otago, Dunedin, New Zealand; 2 Chongqing University of Engineering, Chongqing, China

**Keywords:** ChatGPT, EAP, EAP writing, generative artificial intelligence, L2 learner engagement, L2 writing

## Abstract

While a growing body of research has examined the affordances and effects of generative artificial intelligence (GenAI) in language learning, less attention has been paid to how learners engage with these tools during the writing revision process across behavioural, cognitive, and affective dimensions. This qualitative multiple-case study investigates how eight English for Academic Purposes (EAP) learners engage with ChatGPT-4.0 while revising academic writing. Data included screen recordings of learner–AI interactions, interaction transcripts, stimulated recall sessions, and semi-structured interviews. The findings reveal that learner engagement is heterogeneous and multidimensional. Behavioural and cognitive engagement ranged from deliberate, evaluative processing to more mechanical uptake, including instances of overreliance on AI-generated feedback. Affective engagement was similarly mixed: while participants valued the immediacy and accessibility of feedback, some reported a lack of social and contextual support compared to teacher guidance. These findings highlight the importance of teacher mediation and critical AI literacy in supporting meaningful engagement with GenAI. The study contributes to emerging research on AI-assisted language learning by specifying the conditions under which GenAI feedback may foster, rather than undermine, reflective and critical engagement in EAP writing.

## 1 Introduction

The advancement of Generative Artificial Intelligence (GenAI) has transformed the way students learn (Du and Daniel, 2024; Liu and Ma, 2024; Kohnke et al., 2023). GenAI techniques have been around for a while, and the introduction of ChatGPT sparked a flurry of debate among academic groups, internet forums, and the media (Mahapatra, 2024). Due to its adaptability, ChatGPT, one of these tools, has garnered enthusiasm and attention since its introduction (Cotton et al., 2024; Lo et al., 2024).

Regarding academic writing, ChatGPT 4.0 (hereafter ChatGPT) can be a useful tool in the writing field for students during the initial stages of drafting their writing outlines (Imran and Almusharraf, 2023; Salam, 2025; Wang et al., 2024). It proves especially beneficial in facilitating L2 writing preparatory tasks and activities before formal writing, such as generating ideas for essays (Cingillioglu, 2023; Du, 2025; Fitria, 2023), outlining key points (Lingard, 2023; Moorhouse and Kohnke, 2024), summarising previous writers’ perspectives (Ray, 2023), and promoting reflective thinking throughout the whole writing process (Chen et al., 2022; Su et al., 2023). Instead of being a learning assistance tool, ChatGPT may be used as a performance support system (Meishar-Tal, 2024). Meishar-Tal (2024) argues that the primary goal of students using this tool is to enhance their cognitive and writing abilities, rather than attempting to bypass cognitive information processing mechanisms as a shortcut to blindly and quickly produce writing products. While ChatGPT offers the aforementioned advantages, the academic community has raised several concerns regarding its use, sparking intense debates on whether it should be incorporated into writing classrooms (Vetter et al., 2024). One of the most discussed concerns is whether students will submit ChatGPT-generated content directly as their assignments, potentially leading to accusations of plagiarism (Perkins et al., 2024). Another major concern is the potential for academic unfairness. Early studies (e.g., Cingillioglu, 2023; Chaka, 2024) have primarily focused on the issue of plagiarism in ChatGPT-generated texts and the degree of detection and evaluation of these texts.

Recent research on ChatGPT has largely foregrounded ethical concerns such as academic integrity, authorship, and potential misuse in educational contexts (Pack and Maloney, 2023). More broadly, the rapid integration of GenAI in language education has raised important questions about the responsible use of GenAI-generated content and the need for clear institutional policies to guide ethical AI-assisted learning practices (Sallam and Sallam, 2025). As highlighted in recent research, educational institutions are increasingly expected to develop transparent guidelines and pedagogical strategies that promote responsible engagement with GenAI while maintaining academic standards. While these discussions provide valuable insights into the ethical governance of GenAI in education, existing research has paid comparatively little attention to how learners actually interact with GenAI tools during the process of academic writing. In particular, few studies have examined learners’ behavioural actions, cognitive sense-making, and affective responses as they engage with GenAI-generated feedback while drafting and revising academic texts. This lack of fine-grained, process-oriented evidence limits our understanding of how ChatGPT mediates learning processes rather than merely influencing writing outcomes.

The present study investigates how eight Chinese EAP students engage with ChatGPT while seeking support for writing and revision tasks in a private higher education institute in China that provides English-medium academic preparation for students intending to pursue undergraduate or postgraduate study abroad. The private institute places particular emphasis on intensive writing practice and formative feedback, creating a learning environment in which students frequently seek additional feedback resources to refine their academic writing. By examining EAP students’ interactions with ChatGPT, this study provides a process-oriented account of how GenAI-mediated feedback shapes behavioural, cognitive, and affective engagement, extending existing conceptualisations by identifying distinctive engagement patterns.

## 2 Review of the literature

### 2.1 ChatGPT in L2 writing

ChatGPT has become increasingly prominent in L2 writing research because it can support multiple stages of the writing process, including brainstorming, drafting, feedback reception, and revision (Ab Hamid et al., 2023; Lingard, 2023). Compared with earlier automated writing tools that were largely limited to lower-order correction, ChatGPT has attracted particular attention because of its dialogic interface, contextual responsiveness, and capacity to provide individualised feedback (Escalante et al., 2023; Pavlova, 2024). As a result, it is increasingly being conceptualised not simply as a text generator but as a mediating tool that may reshape how learners engage with writing tasks, process feedback, and make revision decisions (Koltovskaia et al., 2024; Yan and Zhang, 2024).

Recent empirical studies suggest that ChatGPT can make meaningful contributions to L2 academic writing. Hongxia and Razali’s (2025) quasi-experimental study found that ChatGPT was associated with improvement across multiple dimensions of English academic writing, including content, structure and coherence, grammar, vocabulary, spelling, and form, while many learners also reported positive effects on engagement. Similarly, Wang and Ren (2024) mixed-method study in a linguistics course showed that students used ChatGPT not only for linguistic support but also to shape discourse, organise information, and support collaborative multimedia academic writing. This suggests that GenAI may function as a broader composing resource rather than merely an editing tool.

At the same time, the literature increasingly shows that ChatGPT’s pedagogical value cannot be treated as straightforward or uniformly positive. Koltovskaia et al. (2024) explicitly argue that there has been limited attention to how students actually use ChatGPT for text revision, and their study demonstrates that learners’ engagement with ChatGPT during revision is behaviourally, cognitively, and affectively differentiated rather than stable or uniform. Yan and Zhang (2024) make a similar point in their mixed-method multiple case study, showing that learner engagement with ChatGPT-generated corrective feedback is shaped by individual differences, technological competence, and the ability to regulate interaction with the tool. Their findings suggest that even in apparently interactive AI-supported writing environments, effective learning cannot be assumed. It depends on how learners process, evaluate, and act on feedback.

This tension becomes even clearer when ChatGPT is compared with more established automated writing evaluation systems. Shi et al. (2025) found that although ChatGPT outperformed AWE and control conditions in post-writing performance, students in the ChatGPT condition reported a weaker ideal L2 writing self than those in the AWE group, and qualitative data pointed to overreliance on the tool together with a loss of creativity and agency. In other words, stronger textual performance did not automatically translate into more desirable writing-related self-development. A comparable caution appears in Wang and Ren (2024) study, where the authors acknowledge the need for clear pedagogical guidance so that ChatGPT use enhances rather than displaces students’ authentic voice and responsible academic decision-making. These studies suggest that the key issue is no longer whether ChatGPT can assist L2 writing in general. The more important question is how its support is mediated through learners’ engagement with the writing process itself.

Many empirical studies suggest that ChatGPT can support L2 writing by facilitating idea generation, linguistic refinement, and iterative revision processes (e.g., Barrot, 2023; Kasneci et al., 2023). In particular, they have highlighted the potential of ChatGPT to provide immediate, accessible feedback that may encourage learners to engage more actively with drafting and revising their texts (Moorhouse and Kohnke, 2024; Wang et al., 2024). At the same time, this emerging literature also raises concerns that such support may not always translate into meaningful learning. Without sufficient critical evaluation, learners may use GenAI-generated suggestions uncritically or rely on the tool in ways that reduce cognitive effort, potentially limiting opportunities for developing independent judgment and authorial control (Kasneci et al., 2023; Shi et al., 2025).

Two limitations remain particularly relevant. First, much of the existing work focuses on perceived usefulness, writing performance, or general learner attitudes toward AI tools (e.g., Guan et al., 2025; Wang et al., 2024; Valova et al., 2024), with comparatively fewer studies providing detailed accounts of how learners interact with AI-generated feedback during the revision process itself. As a result, the micro-level processes through which learners interpret, evaluate, and act on such feedback remain underexplored. Furthermore, although learner engagement is increasingly recognised as central to understanding technology-supported learning (Fredricks et al., 2004; Greene, 2015), relatively few studies examine engagement in a process-oriented manner across its behavioural, cognitive, and affective dimensions. Existing research often treats engagement as a general outcome or self-reported construct, rather than analysing how it unfolds dynamically during interaction with ChatGPT. This points to a need for more fine-grained, qualitative investigations that capture how learners engage with GenAI-generated feedback in real time and across multiple dimensions of the learning process.

### 2.2 Engagement as a multi-dimensional construct

Student engagement has long been recognised as a multidimensional construct that captures the ways learners participate in and respond to learning activities. According to Fredricks et al. (2004), engagement encompasses three interrelated dimensions: behavioural, cognitive, and affective engagement. Behavioural engagement refers to students’ observable participation in learning activities, such as effort, persistence, and task involvement. Cognitive engagement concerns the psychological investment learners make in understanding content, including the use of strategies for problem-solving, reflection, and self-regulation (Ellis, 2010). Affective engagement relates to students’ emotional reactions to learning activities, such as interest, enjoyment, anxiety, or frustration. This tripartite framework provides a comprehensive lens for examining how learners interact with emerging educational technologies, including generative artificial intelligence tools.

Within the context of GenAI–assisted learning, this framework is particularly useful for understanding how tools such as ChatGPT mediate learners’ engagement with writing tasks. Behavioural engagement can be observed in the ways students actively interact with ChatGPT while completing academic tasks, including prompting the system, revising drafts, and incorporating generated feedback. A growing body of research suggests that students frequently use ChatGPT to support writing-related activities such as generating outlines, brainstorming ideas, and proofreading drafts (Barrot, 2023; Chan and Hu, 2023; Du and Kohnke, 2025; Liu and Ma, 2024). Studies have also shown that learners employ the tool to complete or refine writing assignments more efficiently (Alsaweed and Aljebreen, 2024; Zhao, 2025). These findings indicate that GenAI can stimulate active task participation and iterative revision processes, thereby fostering behavioural engagement in writing tasks.

However, the literature also highlights important limitations of GenAI that may shape learners’ engagement with the tool. Concerns have been raised regarding the reliability and quality of GenAI- generated outputs, including issues such as repetition, inconsistency, unclear explanations, and the potential risk of plagiarism. For example, Alsaweed and Aljebreen (2024), using a mixed-methods design combining quantitative and qualitative analyses, found that ChatGPT performed effectively in correcting several categories of ESL writing errors, including verb forms, word order, passive voice, conditional sentences, subject–verb agreement, singular and plural nouns, word morphology, and word choice. Nevertheless, its performance was only moderate in areas such as article use, modal verbs, and verb tenses, and weaker in aspects related to clarity, connectors, sentence structure, and non-idiomatic expressions. Similarly, Van Niekerk et al. (2024) highlighted concerns regarding the accuracy of GenAI-generated references, limited critical insight, and potential issues with objectivity. Such limitations may influence how learners evaluate and respond to GenAI-generated feedback, potentially affecting their trust in the tool and shaping the depth of their engagement. These findings suggest that while GenAI tools can encourage active participation in writing tasks, learners must also engage critically with GenAI-generated feedback. Understanding how students navigate these opportunities and limitations requires a closer examination of their behavioural actions, cognitive processing, and affective responses during interaction with GenAI systems. Applying the behavioural–cognitive–affective engagement framework, therefore, offers a useful theoretical lens for exploring how learners meaningfully engage with ChatGPT while drafting and revising academic texts.

Much of the literature conceptualises cognitive engagement as a multidimensional construct encompassing learners’ investment in deep processing, strategic regulation, and sustained attention during learning tasks (Fredricks et al., 2004; Greene, 2015). Within this perspective, cognitive engagement is closely associated with self-regulated learning (SRL), as it reflects learners’ capacity to plan, monitor, and evaluate their cognitive processes in goal-directed activity (Pintrich, 2000; Zimmerman, 2002). Rather than being a static trait, cognitive engagement is enacted through observable regulatory behaviours, such as revising ideas, evaluating alternative solutions, and integrating new information with prior knowledge (Greene, 2015; Schunk and Greene, 2018). In this sense, self-regulation constitutes a central mechanism through which cognitive engagement is operationalised and made visible in learning contexts. A core dimension of cognitive engagement is metacognitive regulation, which involves learners’ ability to monitor their understanding, control mental effort, and strategically adjust their learning approaches (Azevedo and Cromley, 2004; Winne and Hadwin, 2010). Metacognitive engagement is particularly critical in complex tasks such as L2 academic writing, where learners must continuously evaluate linguistic accuracy, rhetorical structure, and argument coherence. Existing research suggests that environments which provide timely, process-oriented support can facilitate such metacognitive activity by prompting learners to reflect on their decisions and regulate their cognitive effort more effectively (Azevedo and Cromley, 2004; Bannert, 2009). Importantly, cognitive engagement should therefore not be reduced to effort or task completion alone, but understood as an iterative process of evaluation, monitoring, and strategic adjustment.

Within the emerging literature on ChatGPT, there is growing evidence that tools such as ChatGPT may reshape how cognitive and metacognitive engagement is enacted. Several studies suggest that ChatGPT can support aspects of self-regulated learning by providing immediate, iterative feedback that prompts learners to reflect on their language use and revise their texts (Ng et al., 2024; Wu et al., 2024). However, this support is not uniformly beneficial. Yan (2024), in a mixed-methods study of L2 writers’ feedback-seeking behaviours, found that interacting with ChatGPT often requires a heightened level of metacognitive awareness. Learners actively articulated evaluative judgments, negotiated the relevance of ChatGPT-generated suggestions, and integrated external feedback with their own internal standards. This finding extends earlier models of feedback-seeking behaviour, which conceptualise it primarily as a cost–benefit evaluation process (Ashford et al., 2003), by demonstrating that AI-mediated feedback environments can involve more complex forms of metacognitive engagement.

Affective engagement is commonly understood as learners’ emotional responses to learning activities, including feelings such as interest, enjoyment, anxiety, and frustration, which influence their willingness to participate and persist (Fredricks et al., 2004; Bond and Bergdahl, 2022). ChatGPT significantly promoted knowledge sharing among students, fostering a collaborative and participatory learning environment in terms of positive emotional engagement (Duong et al., 2023). Moreover, Wu et al. (2024) found that during the process of seeking feedback, students experienced a significant anxiety reduction. ChatGPT effectively alleviated student anxiety by removing fears related to unresolved issues. Similarly, Bond and Bergdahl (2022) and Rad and Rad (2023) argue that anxiety plays an important role in affective engagement and is a key indicator of this emotional dimension. Additionally, our preliminary review of the literature indicates that while using ChatGPT may negatively affect social interaction, the majority of students’ interactions with ChatGPT are highly positive, with many students describing ChatGPT as providing emotional support in their independent learning process. For instance, Shaikh et al. (2023) reported that participants’ satisfaction scores indicated a positive experience with ChatGPT. Similarly, El Khodr et al. (2023) found that ICT students in Case Studies 1 and 2 generally reported enjoying the experience of using ChatGPT. Likewise, Ab Hamid et al. (2023) noted that students appreciated and enjoyed ChatGPT’s feedback and enjoyed how it stimulated meaningful discussions and allowed students to explore different perspectives on information. The literature suggests that enjoyment is widely recognised as a central component of affective engagement, serving as a key indicator of learners’ emotional responses during interaction with ChatGPT’s feedback. At the same time, existing studies report predominantly positive affective responses to ChatGPT, with learners expressing satisfaction and emotional support, although such interactions may also have implications for reduced social engagement in learning contexts.

### 2.3 Present study and research questions

GenAI tools such as ChatGPT create an interactive, dialogue-based feedback environment that enables learners to engage with writing feedback in iterative and potentially more active ways. Rather than passively receiving corrections, EAP learners can request clarification, pose follow-up questions, and refine their writing through ongoing interaction. In this sense, feedback becomes a dynamic process involving not only the reception of information but also its interpretation, evaluation, and application. Such interactional affordances are particularly relevant in EAP contexts, where learners often require timely, individualised, and context-sensitive feedback to support the development of academic writing. A great deal of research has examined the affordances of ChatGPT for L2 writing, including its effects on writing quality, learner perceptions, and feedback processes. However, less attention has been paid to how learners engage with GenAI-generated feedback during the revision process itself. In particular, there remains limited multiple qualitative evidence capturing how behavioural, cognitive, and affective dimensions of engagement unfold in interaction with generative AI. Addressing this gap, the present study adopts the triadic framework of learner engagement (Han and Hyland, 2015) to examine how eight Chinese EAP learners interact with ChatGPT-generated feedback during academic writing revision. By focusing on engagement as a multidimensional and process-oriented construct, this study moves beyond evaluating tool effectiveness to explore how learners regulate and experience GenAI-mediated feedback. In doing so, it contributes to a more nuanced understanding of the conditions under which ChatGPT may support or constrain meaningful engagement in the process of EAP writing revisions. The study is guided by the following research question:

*RQ:* How and why do EAP learners exhibit different behavioural, cognitive, and affective engagement patterns when interacting with ChatGPT during revision?

## 3 Methods

### 3.1 Research design and participants

At the outset of the study, all participants (see Table 1) attended an internal seminar entitled “Generative AI Has Revolutionised and Transformed EAP Writing”, organised by a private school. Eight participants were included in this qualitative multiple-case study. To further protect participant confidentiality, detailed individual demographic profiles are not reported. Participants were learners enrolled in the institute’s EAP programme and represented a range of disciplinary backgrounds. Their ages ranged from 19 to 29 years, and the sample included both undergraduate- and postgraduate-level learners. English proficiency ranged from upper-intermediate to advanced, based on institutional placement records. Several participants reported prior experience with AI-supported writing or search tools, while others had little or no previous experience. The seminar was delivered during participants’ EAP writing course and aimed to introduce the core writing-related functions of ChatGPT, its basic operational features, and potential pedagogical applications for EAP writing tasks. The session was designed to familiarise students with the tool in preparation for EAP writing assessments and to support their L2 academic writing development.

**Table 1:** Participants’ profiles.

Variable	Category
Sample size	8
Age range	19–29
Degree level	Undergraduate / Postgraduate
Discipline grouping	Business, STEM, Health, Humanities/Social Sciences
English proficiency	Upper-intermediate to Advanced
Prior AI experience	Some prior experience / little or no prior experience

**Figure 1:**
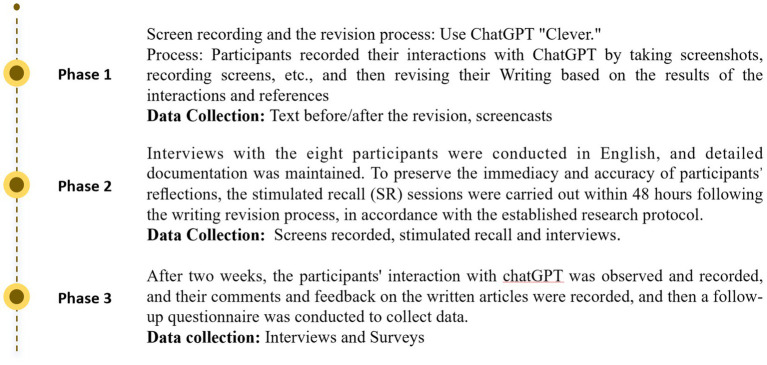
Three steps of workshop data collection.

The one-hour seminar was organised and delivered by the first author in English to introduce participants to the basic functions and pedagogical uses of ChatGPT for academic writing. The first 40 min focused on demonstrating the core functionalities of ChatGPT, including prompt formulation strategies, practical usage techniques, and information about upgrading to ChatGPT-4. The remaining 20 min consisted of an interactive question-and-answer session during which participants raised practical questions and engaged in demonstrations on how to save interaction records, capture screenshots, and selectively incorporate AI-generated responses into their writing tasks. Participants were subsequently recruited using a purposive sampling approach, which is widely employed to enable an in-depth exploration of participants’ experiences and perspectives (Campbell et al., 2020). Recruitment targeted students enrolled in the institute’s EAP programme who had prior experience using ChatGPT or expressed interest in using ChatGPT to support their academic writing. To ensure the relevance of the data, the inclusion criteria required participants to (1) be currently enrolled in the EAP writing course, (2) have intermediate to advanced English proficiency, and (3) be willing to use ChatGPT as a feedback resource during the writing revision process while allowing their interactions to be recorded for research purposes. Finally, eight participants were selected for the study. This relatively small sample size was considered appropriate for the process-oriented design of the research, which aimed to generate rich, detailed data on learners’ engagement with ChatGPT-mediated feedback rather than to achieve statistical generalisation. The number of participants also enabled close observation of individual interaction processes through screen recordings, stimulated recall sessions, and follow-up interviews. Following the seminar, data collection was conducted in three phases (see Figure 1).

For the research design, this qualitative multiple-case study consisted of two parts (see 3.2 and 3.3). The first part focused on examining the participants’ recorded videos and written texts, while also referring to the subsequent stimulated recall sessions and interviews to explore and gain deeper insights into their behavioural engagement. The second part analysed participants’ video screenshots, stimulated recall data, and interview transcripts in order to assess their cognitive and affective engagement. Representative screenshots and interview excerpts were selected and presented in the Results section.

### 3.2 Data collection

Data collection was conducted over three sequential phases to capture learners’ behavioural, cognitive, and affective engagement with ChatGPT during the EAP writing revision process (see Figure 1).

#### 3.2.1 Phase 1: Screen recording (May, 2025)

The first phase took place during the participants’ regular EAP writing course when they were preparing for their EAP examinations and typically spent more than eight hours per day on academic study. Before beginning the task, the eight participants watched a tutorial video explaining how to use Tencent Video to record their screens, store recordings in the cloud, and manage video files. This preparation ensured that all participants were familiar with the recording procedures before data collection. Participants then independently revised their EAP writing assignments using ChatGPT as a feedback resource while their screens were recorded. The screen recordings captured the entire interaction process, including participants’ prompts to ChatGPT, the ChatGPT-generated feedback received, and the subsequent revisions made to their drafts. In addition, participants’ initial writing drafts, ChatGPT interaction logs, and final revised essays were collected. These materials provided detailed evidence of learners’ observable actions during the revision process and were primarily used to analyse behavioural engagement, such as how students prompted ChatGPT, responded to feedback, and modified their texts.

#### 3.2.2 Phase 2: Stimulated recall and semi-structured interviews (Cognitive and affective engagement) (May, 2025–July, 2025)

The second phase focused on exploring participants’ cognitive processes and emotional responses during their interactions with ChatGPT. Delayed stimulated recall (SR) sessions were conducted within 48 h after the revision activity to ensure that participants could accurately recall their thought processes and decision-making during the interaction (Gass and Mackey, 2000). During the SR sessions, participants watched the full recordings of their own writing revision process and were asked to comment on their reasoning, interpretations of the feedback, and decisions about whether and how to incorporate ChatGPT’s suggestions. Following the stimulated recall sessions, semi- structured interviews were conducted in English to further explore participants’ experiences, perceptions, and emotional reactions to ChatGPT-generated feedback. All sessions were audio- recorded and transcribed for analysis. These datasets provided insight into learners’ cognitive engagement (e.g., interpretation, evaluation, and decision-making regarding feedback) as well as affective engagement (e.g., confidence, satisfaction, or frustration during the interaction).

#### 3.2.3 Phase 3: Follow-up interview (Longer-term perceptions and engagement patterns) (August, 2025- September, 2025)

The final phase involved a semi- structured interview administered two weeks after the main data collection period. By this time, participants had gained more experience using ChatGPT and had become familiar with prompting strategies while completing additional writing tasks. The purpose was to capture participants’ longer-term reflections and emerging usage patterns related to ChatGPT. The interview contained ten items addressing participants’ overall attitudes toward using ChatGPT for EAP writing, perceived benefits and limitations, preferred prompting strategies, approaches to evaluating ChatGPT-generated feedback, and views on ethical considerations related to ChatGPT use in academic contexts.

### 3.3 Data analysis and coding

The data analysis involved a systematic examination of the screen recordings, original and revised texts, stimulated recall (SR) transcripts, and semi-structured interview records. All video interactions and audio recordings were fully transcribed, and the transcripts were manually cross-checked by all the authors against the original recordings to ensure accuracy. To illustrate how revision episodes were documented and aligned across data sources during analysis, Table 2 provides a very detailed example of the analytical record used to track selected participants’ revision operations and strategies.

**Table 2:** Example analysis of participants’ revision operations.

Time	Original text	ChatGPT comments				Revision operations	Revision strategies
		Comments	Suggestions	Logic	Accuracy		
2:15	First and foremost, when facing challenges, people always work as hard as they can. Challenges and difficulties would bring out the best in people. Such examples abound in life. For example, Yangyang, now a well-known blind singer in China, gained fame after he sang a song last year in a big show on the Chinese Spring Festival. Ten years ago, he had a bad car accident and lost his eyesight since.	Overall, the revised paragraph is more logically structured and flows better, making it easier for readers to understand.	“Ten years ago, he had a bad car accident and lost his eyesight since.” → Rewritten as “Ten years ago, he was involved in a serious car accident that resulted in the loss of his eyesight.” This makes the sentence clearer and more grammatically correct	“Such examples abound in life.” → This was adjusted to “There are numerous examples of this in real life” to sound more natural and more logical.	“Challenges and difficulties would bring out the best in people.” → The phrase was reworded to avoid the future tense (“would”) and ensure it reads as a general statement.	Revised paragraph: First and foremost, when faced with challenges, people often strive to overcome them with all their might. Difficulties can bring out the best in individuals, pushing them to exceed their limits. One such example is Yangyang, a well-known blind singer in China, who gained widespread fame after performing a song on a popular Chinese Spring Festival television show last year. Ten years ago, he was involved in a serious car accident that resulted in the loss of his eyesight. Despite this, he persevered and turned his adversity into an opportunity to showcase his talent.	Good grammar detection, prompt is observed
5:22	Most young adults prefer to have a separate or independent life from their parents or families as soon as possible. This is because they have a strong urge for freedom in doing what they desire.	Overall, the revised paragraph is clearer, more cohesive, and grammatically sound, enhancing readability and academic tone.	“Prefer to have a separate or independent life” → Rewritten as “seek independence” to make the sentence more concise and focused.	The revised version maintains logical flow, with the first sentence clearly introducing the preference for independence, and the second sentence explaining why this preference exists, with more precise language.	This is because they have a strong urge for freedom in doing what they desire.” → Reworded to “This desire for autonomy is driven by their strong need for freedom and the ability to make their own choices.” This revision clarifies the cause and enhances the flow of ideas. It also avoids the awkward phrasing “urge for freedom in doing what they desire,” which can be simplified.	Most young adults seek Independence from their parents or families as soon as possible. This desire for autonomy is driven by their strong need for freedom and the ability to make their own choices.	Very excellent revision update

The data were analysed using an iterative thematic analysis informed by the triadic engagement framework (behavioural, cognitive, affective), with explicit procedures to ensure transparency and traceability across data sources. Given that the dataset combined real-time interactional data (screen recordings and texts) with retrospective data (stimulated recall and interviews), the unit of analysis was defined differently for each source and explicitly operationalised prior to coding. For the screen-recorded data, the unit of analysis was a revision episode, defined as a bounded interaction sequence consisting of: (a) a learner prompt, (b) ChatGPT’s generated feedback, and (c) the learner’s observable response (e.g., revision, no revision, or follow-up query). Please see Supplementary Appendix A: Various Stages of the Revision Process and SR for an example. Each episode was assigned a unique identifier and logged in a structured dataset that included timestamp, original text, ChatGPT feedback, and revision outcome. For example, a revision episode in which a learner prompted ChatGPT to revise a sentence, received a suggestion (e.g., replacing “nice” with “thoughtful,” see Table 3), and then edited the text accordingly was treated as one analytical unit. For the SR and interview data, the unit of analysis was a meaning segment, defined as a coherent stretch of discourse in which participants explained or reflected on a specific revision decision, feedback interpretation, or experience of using ChatGPT. These segments were identified by all the authors through repeated reading and were linked, where possible, to corresponding revision episodes in the interactional dataset.

**Table 3:** P7’s comments regarding words choices.

Video time	Original	ChatGPT comments	P7’s SR
4:22	High pay means that I could provide a better life for my family – a good home, plenty of food, nice clothes, nice birthday presents and money for my children’s education.	The word “nice” is a bit vague and informal. In an academic context, it’s better to choose more specific adjectives that provide clearer meaning. Suggested revision (as already shown above): “thoughtful birthday gifts” instead of “nice birthday gifts.”	Accepted.

Specifically, the analysis proceeded in two coding cycles. In the first cycle, the authors conducted inductive coding across the entire dataset. Codes were assigned line-by-line to capture three types of data: (1) observable revision actions (e.g., “accepted suggestion,” “ignored feedback,” “requested clarification”), (2) cognitive processes (e.g., “compared with prior knowledge,” “questioned accuracy,” “interpreted meaning”), and (3) affective responses (e.g., “felt reassured,” “expressed frustration,” “reported anxiety”). For instance, when a participant stated in SR, “I think this suggestion is not correct because I’ve seen examples using dashes,” this segment was initially coded as “reject suggestion” (behavioural).

In the second cycle, the initial codes were systematically reviewed and reorganised. This involved (a) merging conceptually similar codes (e.g., “copying suggestion” and “direct insertion” into “accept”), (b) splitting overly broad codes (e.g., separating “evaluation” into “comparison” and “judgement”), and (c) removing low-frequency or weakly supported codes that did not recur across cases. The refined codes were then mapped onto the three engagement dimensions based on explicit criteria: observable actions were categorised as behavioural engagement; acts of interpretation, comparison, and judgement as cognitive engagement; and expressions of emotion or attitude as affective engagement. This process resulted in a structured coding framework that distinguished between, for example, different forms of uptake (e.g., immediate acceptance vs. modified incorporation), types of cognitive processing (e.g., comparison vs. questioning), and varied affective responses (e.g., reassurance, enjoyment, frustration, concern).

To enhance trustworthiness, the authors each coded the previously mentioned error categories on their own, then compared their coding frameworks and settled any discrepancies. This review focused on three aspects: (1) the clarity and consistency of code definitions, (2) the appropriateness of code application to specific data excerpts, and (3) the validity of code categorisation within the three engagement dimensions. Discrepancies (e.g., whether a segment reflected behavioural action or cognitive evaluation) were discussed and resolved through iterative comparison and refinement of the coding scheme. All revisions to code definitions and category boundaries were documented to maintain an audit trail. Given the exploratory, small-scale qualitative design, rigour was established through (a) explicit definition of analytical units, (b) systematic and replicable coding procedures, (c) cross-data triangulation, and (d) collaborative review of coding decisions. The final coding framework, including code definitions and representative examples, is presented in Supplementary Appendix C.

### 3.4 Ethical consideration

Ethical approval for this study was obtained from the corresponding author’s Institution. All participants were provided with detailed information about the purpose of the study, the procedures involved, and their rights as research participants. Written informed consent was obtained from each participant prior to data collection. Participants were assured that their participation was entirely voluntary and that they could withdraw from the study at any time without consequence. To protect participant confidentiality, all identifying information was removed, and pseudonyms were used throughout this report. All data, including screen recordings, interview transcripts, and ChatGPT interaction logs, were stored securely on password-protected university servers accessible only to the research team. Data will be retained for five years following publication, after which it will be securely deleted in accordance with institutional data protection policies.

## 4 Results

### 4.1 Behavioural engagement

The analysis of screen recordings, interaction logs, and revised drafts showed that behavioural engagement was evident across all eight learners, although its form and intensity varied. In this study, behavioural engagement was operationalised as observable learner actions during interaction with ChatGPT, including prompting, reviewing, comparing, and revising. Uptake was treated as a related but more specific indicator, referring to whether and how individual ChatGPT suggestions were incorporated into revised drafts.

As shown in Table 4, all participants accepted more suggestions than they rejected across both higher-order concerns (HOCs) and lower-order concerns (LOCs). Each count represents one discrete ChatGPT suggestion that was either incorporated into the revised text (accepted) or not implemented (discarded). While the overall pattern indicates a higher frequency of acceptance, the volume and type of uptake varied across participants. Lower-order feedback was taken up more frequently than higher-order feedback, with most accepted suggestions relating to local revisions such as grammatical accuracy, clarity, and phrasing. These uptake counts provide a descriptive account of revision outcomes, reflecting observable changes made to the text.

**Table 4:** Summary of participants uptake decisions across HOC and LOC.

Participant	HOC accepted	HOC discarded	LOC accepted	LOC discarded	Total accepted	Total discarded	Total suggestions
P1	25	0	6	14	31	14	45
P2	20	6	1	6	21	12	33
P3	20	2	12	5	32	7	39
P4	20	1	27	8	47	9	56
P5	11	4	19	4	30	8	38
P6	12	1	12	1	24	2	26
P7	9	1	20	7	29	8	37
P8	2	3	30	7	32	10	42

In addition to these uptake patterns, the interaction data showed that EAP learners engaged in a sequence of actions before revision, including reviewing ChatGPT’s suggestions and comparing them with their original drafts. Across six participants, revisions were often made in response to feedback that was perceived as relevant to the intended meaning of the text. For example, in P7’s revision of “nice birthday gifts” to “thoughtful birthday gifts,” the change followed a system-generated comment that the original wording was vague in an academic context. Similar instances were observed across multiple participants, where revisions were carried out following interaction with ChatGPT suggestions. Detailed uptake decisions by feedback category are provided in Supplementary Appendix B.

The data show that different patterns of uptake accompanied learners’ observable actions during revision. Across cases, instances were identified in which multiple ChatGPT suggestions were incorporated into revised drafts. These instances were reflected in the frequency of accepted suggestions reported in Table 4 and Supplementary Appendix B.

At the same time, the data also included instances in which suggestions were accepted with minimal modification or without explicit evidence of comparison with the original text in the interaction records. For example, Sara described her revision process as follows:

“*…and all I did was pay for a premium subscription and click* ‘*accept, accept*’ *for everything. This process was meaningless because I would inevitably make the same mistakes in my next writing session.”* (*P6*’*s SR*)

Such instances were observed alongside cases where learners reviewed suggestions and made targeted revisions following interaction with the system. These observations indicate that accepted suggestions correspond to a range of observable revision outcomes across participants. These uptake patterns are reported descriptively and do not indicate the underlying level of cognitive or evaluative processing involved in each case.

### 4.2 Cognitive engagement

Analysis of stimulated recall (SR) and interview data showed variation in how learners processed, evaluated, and used ChatGPT-generated input during writing revision. Across the dataset, instances were identified in which learners questioned, rejected, or incorporated AI suggestions. These actions were reflected in participants’ verbal reports and interaction records.

In several cases, learners compared ChatGPT’s suggestions with their prior linguistic knowledge, genre awareness, or previous writing experience. For example, in response to a suggestion to replace an em dash with a colon, Lawrence rejected the recommendation and referred to examples from EAP writing sources:

*“I think it doesn’t make sense. I always read some EAP L2 writing samples on the official website. They all use dashes there as I do.” (P7’s SR)*

This type of response, involving reference to external sources or prior knowledge, was observed in five of the eight learners. The frequency and extent of such responses varied across participants.

**Figure 2:**
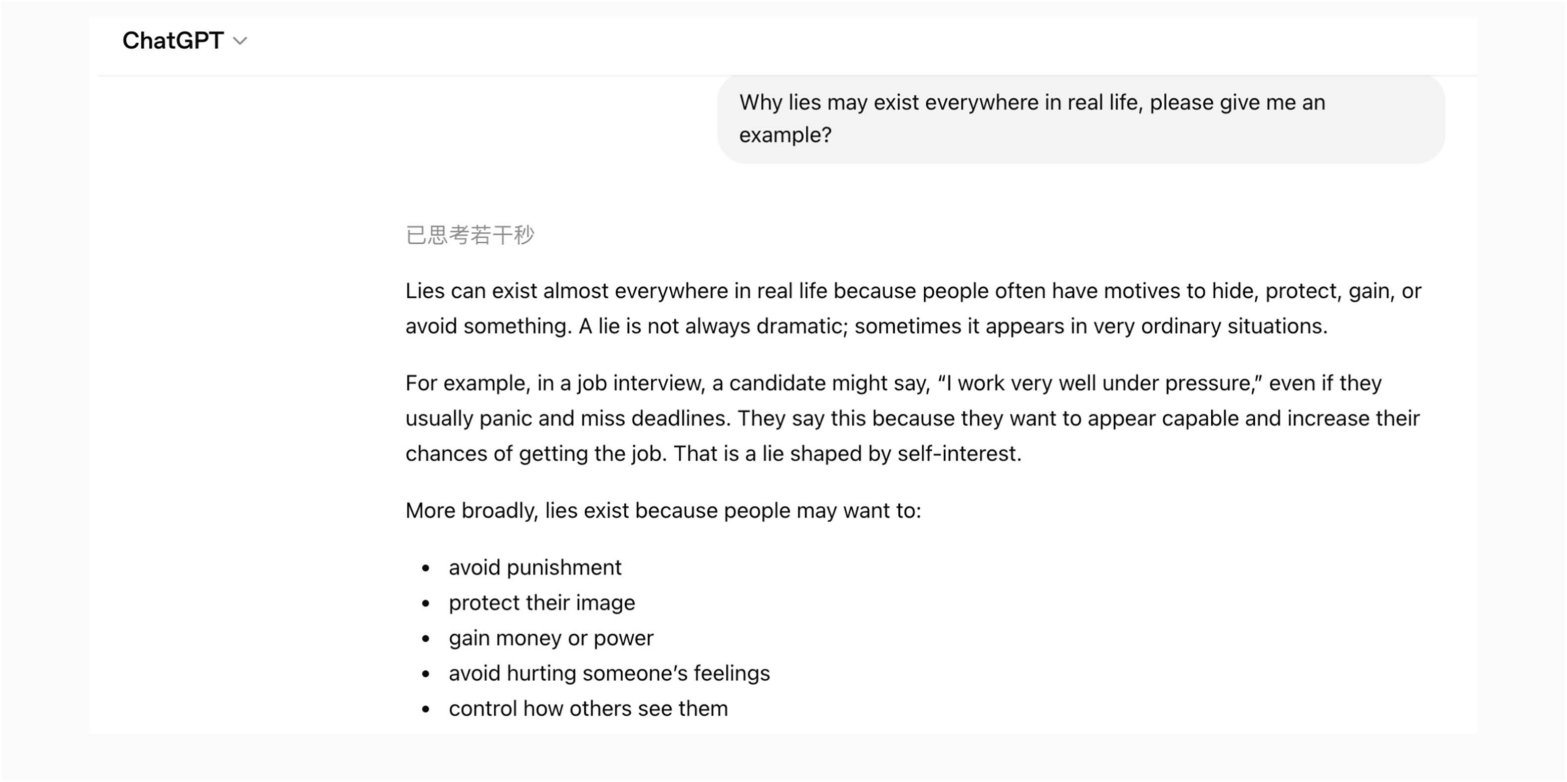
P4’s engagement with ChatGPT.

In addition to rejection and comparison, the data also included instances in which learners used ChatGPT-generated content to support idea development. For example, P4 (see Figure 2 and SR) described using ChatGPT to generate examples for an essay topic:

*“For instance, one of my EAP writing assignments was on the topic ‘Honesty is the best policy.’ I had no idea how to approach it, so I gave ChatGPT a prompt… Its response was really great, as shown in the figure below. It suddenly made me realise that I often say I’m fine in real life, but that doesn’t necessarily mean I really am. That’s a perfect example of a white lie… I really like this example, and suddenly I feel like I already have the materials that I can use for the EAP writing homework.” (P4’s SR)*

In this case, the learner reported using the generated content to form an example connected to personal experience. Similar instances were observed across multiple participants, where GenAI-generated input was incorporated into the writing process at different stages. These observations show that learners engaged in a range of actions when interacting with ChatGPT-generated input, including rejection, comparison, and incorporation. These actions are reported descriptively and reflect observable patterns of interaction and reported processing during the revision process.

### 4.3 Affective engagement

This section’s data was collected by SR and interviews. Analysis of stimulated recall and interview data showed that learners’ affective engagement with ChatGPT was complex rather than uniformly positive or negative. Across participants, a range of affective responses was identified, including expressions of reassurance, frustration, concern, and preference for alternative forms of support. Several participants described positive emotional responses when using ChatGPT during revision. These responses included references to reduced anxiety, increased confidence, and a sense of support when dealing with language-related difficulties. Such responses were reported by P1, P3, P4, P5, P7. For example, P7 stated:

*“ChatGPT’s feedback really gives me emotional value/support. It reduces my anxiety and provides a complete sense of relief, both physically and mentally. At the very least, I’ve gained confidence. I no longer feel alone… With ChatGPT, at least I no longer need to worry about grammar in my writing.” (P7’s SR)*

Similarly, P6 emphasised the emotional reward she associated with the tool’s interactive nature:

*“ChatGPT offers an interactive experience. It treats you like a student, explaining how to revise sentences and ensuring logical coherence and fluency… I find it incredibly helpful and rewarding.” (P6’s SR)*

In addition to these responses, the data also included expressions of frustration and concern. These responses were reported in situations where participants described a mismatch between their intended prompts and the system’s output. For example, P4 noted:

*“Sometimes I feel the prompts I provide don’t align with what ChatGPT delivers… ChatGPT immediately generated a full essay. Structurally, it was almost perfect—or so it seemed to me. But why did I provide two prompts? My first instruction was meant to let ChatGPT provide me with ideas and tips… ChatGPT lacks transparency in how it operates, which leaves me deeply concerned.” (P4’s SR)*

Some participants also reported similar concerns. These responses were expressed by P1, P4, P5, P6, P7. For example, P7 stated:

*“Every time I depend on the ideas from ChatGPT makes me quite concerned about the danger or overreliance on this tool. I lose a lot of sleep over this because one day, if I don't have access to ChatGPT, or I don't have money to pay for the membership, I will fall behind.” (P7’s SR)*

In addition, several participants (P1, P2, P4, P7, P8) referred to their experiences with teacher guidance when describing their use of ChatGPT. For example, P6 stated:

*“The next day, I brought the same topic to my EAP teacher, who guided me with logical, step-by-step inspiration… This step-by-step guidance not only taught me how to complete this particular topic but also prepared me for handling similar ones in the future.” (P6’s SR)*

Across the dataset, EAP learners reported multiple types of affective experiences during interaction with ChatGPT. These findings are presented descriptively and reflect participants’ reported affective responses in relation to their use of the tool.

## 5 Discussion

### 5.1 Revisiting the major findings

Based on the three-dimensional conceptual framework of learner engagement (Han and Hyland, 2015; Zhang and Hyland, 2018), this study explored the interaction of eight Chinese EAP learners with ChatGPT-generated feedback while revising their academic writing. Rather than seeking to prove the overall effectiveness of the tool or measure definitive writing outcomes, this qualitative multiple-case study offers a process-oriented perspective on how students behaviourally, cognitively, and affectively engaged with AI-mediated feedback. The findings highlight that learner engagement with ChatGPT is highly complex and occasionally contradictory. While the tool can provide immediate support for certain writing needs, the observed engagement patterns reveal a dynamic tension between deliberate tool use and the risks of shallow uptake and overreliance.

### 5.2 The complexity of multi-dimensional engagement

From a behavioural perspective, the findings indicate that observable engagement during revision is best understood as a sequence of interactional actions (e.g., reviewing, comparing, and revising). Screencast analysis showed that students spent an average of 57.8 min per revision session. This substantial time investment indicates a high level of observable engagement with ChatGPT-generated feedback. In terms of revision operations, across the eight participants, 246 suggestions were incorporated into revised drafts, and 70 were rejected, providing a descriptive account of revision outcomes. Participants accepted most of ChatGPT’s suggestions—particularly those related to grammatical accuracy, clarity, and phrasing—to improve their EAP writing. They rejected suggestions they considered inaccurate, especially those involving punctuation, demonstrating critical judgment by identifying and challenging these issues.

Importantly, the variation in uptake patterns observed across participants suggests that behavioural engagement is shaped by how learners regulate their interaction with AI-generated feedback. In cases where learners engaged in reviewing and comparing suggestions before revising, uptake appeared to be embedded within a more deliberate sequence of actions. In contrast, instances of rapid or repeated acceptance indicate that the same behavioural outcome (i.e., accepted feedback) can emerge under different conditions, including reduced evaluation or time-oriented decision-making. This distinction highlights a key limitation in treating uptake frequency as a proxy for engagement and supports recent calls to differentiate between observable revision behaviour and underlying engagement processes (Chan and Hu, 2023; Pearson, 2024).

Compared with earlier research, which often assumes a closer alignment between feedback incorporation and learning (e.g., Hyland and Hyland, 2006; Ellis, 2009), the current findings highlight a more complex relationship in GenAI-mediated contexts. The generative and immediate nature of ChatGPT allows EAP learners to incorporate feedback rapidly, but this also introduces the possibility that revision actions may be partially decoupled from evaluative processing. Thus, behavioural engagement is influenced by the way EAP learners manage and control their interactions with ChatGPT-generated content.

From a cognitive perspective, the engagement patterns were similarly varied. Rather than uniformly demonstrating high cognitive investment, participants exhibited different degrees of evaluative processing. Some participants demonstrated deliberate cognitive engagement by carefully comparing the suggested feedback with their original sentences, applying reasoning, and consulting relevant literature to challenge the AI’s output (Foroughi et al., 2024; Wu et al., 2024). However, the study also identified a pattern of reflective but dependent uptake. As evidenced by P4’s reflections, ChatGPT’s capacity to rapidly generate complete texts sometimes shifted part of the idea-generation process to the tool. This reduction in independent intellectual investment reflects a boundary condition of interacting with highly generative tools: without appropriate self-regulation, GenAI may reduce learners’ reliance on their own brainstorming processes, potentially limiting opportunities for independent idea generation and evaluative processing (Cai and Xing, 2023).

From an affective perspective, learners reported mixed responses. On one hand, several participants described feelings of reassurance, relief, and increased confidence due to the immediacy and accessibility of ChatGPT’s feedback (Wu et al., 2024; Rad and Rad, 2023). As Cheng and Liu (2022) note, favourable emotional responses to feedback can enhance learners’ readiness to act on it, thereby facilitating deeper learning. These accounts suggest that the tool could be experienced as a low-stakes, non-judgmental form of support. Aligned well with Koltovskaia et al. (2024) and Yilmaz and Yilmaz (2023) study, students were satisfied with ChatGPT feedback. Also, these positive reported emotions were frequently counterbalanced by heightened concerns about technological dependency. Also, resonating with the findings from Frenkenberg and Hochman (2025), participants explicitly expressed anxiety about overreliance and the potential loss of their academic autonomy as well. Furthermore, rather than proving the absolute instructional superiority of human teachers, the data highlighted a continuing desire for face-to-face interaction. The present study pointed to a strong continuing preference for the empathetic, step-by-step scaffolding associated with teacher guidance, suggesting that some learners continued to value forms of socio-emotional support that they did not always perceive in AI-mediated interactions (Escalante et al., 2023).

While the findings identify distinct behavioural, cognitive, and affective patterns, a key contribution of this study lies in explaining why these patterns emerge. The results suggest that engagement is shaped by the interaction of at least three underlying mechanisms. First, task–tool alignment influences the depth of engagement: when ChatGPT is used for lower-order revisions, learners tend to adopt suggestions more mechanically, whereas higher-order revisions are more likely to trigger evaluative processing. Furthermore, the generative capacity of ChatGPT introduces a form of cognitive delegation, whereby parts of the idea-generation process are offloaded to the tool, reducing cognitive load but also potentially limiting independent reasoning. Finally, learners’ beliefs about AI and their level of critical AI literacy mediate how feedback is interpreted and used, shaping whether engagement remains reflective or becomes dependent. Affective responses are governed by a tension between reassurance and perceived loss of control, which in turn influences learners’ willingness to critically engage with the tool. These mechanisms suggest that engagement in AI-mediated writing is not simply a matter of learner behaviour, but an emergent outcome of the interaction between tool affordances and learner dispositions.

### 5.3 Pedagogical implications

The findings suggest that learner engagement with ChatGPT in EAP writing involves the interaction of behavioural actions, cognitive processing, and affective responses observed in this study. In this context, ChatGPT functioned as a source of feedback that participants engaged with in different ways, but in a responsible manner during revision. Rather than being used as a replacement for teacher feedback, ChatGPT was used exclusively by the participants for revising their work, rather than as a tool for generating text.

Across the dataset, participants described both incorporating and questioning GenAI-generated suggestions, as well as expressing varied emotional responses. These patterns may indicate that, in similar EAP settings, learners could benefit from guidance on how to interpret and use GenAI-generated feedback. For example, some participants referred to the need to compare suggestions with their own knowledge or expectations, suggesting a potential role for instructional support in helping learners navigate such processes (Moorhouse and Kohnke, 2024). However, given the small-scale and context-specific nature of the study, these observations should be interpreted as exploratory rather than prescriptive. The findings also show that some participants referred to teacher guidance when discussing their use of ChatGPT, particularly in situations requiring more structured or context-sensitive support. This may suggest that feedback generated by ChatGPT and teacher input were experienced as serving different functions within the writing process, as also noted in recent discussions of GenAI-supported learning environments (Sallam et al., 2025).

The present study does not aim to generalise beyond its specific context, but to offer preliminary insights into how learners engage with ChatGPT-generated feedback in EAP writing in the context of China. These insights may inform further research and pedagogical exploration of how such tools can be incorporated into instructional practices under appropriate conditions.

### 5.4 Theoretical contribution

Beyond describing patterns of learner engagement, the findings of this study also invite a reconsideration of how engagement is conceptualised in AI-mediated learning contexts. While the behavioural–cognitive–affective framework (Han and Hyland, 2015) provides a useful analytical lens, the present findings suggest that its dimensions require further refinement when applied to interactions with GenAI tools.

First, behavioural engagement, often operationalised in terms of observable participation or frequency of revision activity, does not necessarily correspond to meaningful learning. The identification of mechanical uptake patterns indicates that high levels of observable engagement may, in some cases, reflect compliance or convenience rather than deliberate learning-oriented behaviour. This finding suggests a need to distinguish between productive and non-productive forms of behavioural engagement in AI-assisted writing contexts. Second, the findings extend the notion of cognitive engagement by highlighting the emergence of AI-mediated or tool-dependent cognitive processes. While learners may demonstrate reflective engagement when interacting with AI-generated feedback, the capacity of ChatGPT to generate ideas and textual content can shift parts of the cognitive workload away from the learner. This introduces a new dimension of engagement in which cognitive effort is not invested but partially delegated, raising important questions about the nature of learning in AI-supported environments. Third, affective engagement in this study was characterised not by stable positive or negative orientations, but by ambivalence and tension, particularly between reassurance and anxiety, or support and dependency. This suggests that affective engagement in GenAI contexts may be better understood as a dynamic and relational construct, shaped by learners’ ongoing negotiation of trust and perceived control (Table 5).

**Table 5:** P7’s punctuation use in writing.

Video time	Original	ChatGPT comments	P7’s SR
2:18	High pay means that I could provide a better life for my family – a good home, plenty of food, nice clothes, nice birthday presents and money for my children’s education.	The em-dash here is used correctly, but in academic writing, you may want to use it sparingly. If you prefer a more formal tone or academic style, consider using a colon (:) instead. A colon often introduces an explanation or elaboration in academic writing.	I think it does not make sense. I always read some EAP L2 writing samples on the official website. They all use dash there like I do.

These findings suggest that engagement in AI-mediated writing is qualitatively reconfigured. Rather than treating behavioural, cognitive, and affective engagement as stable and separable dimensions, the results point to the need for a more nuanced conceptualisation that accounts for the ways in which GenAI reshapes the depth, distribution, and meaning of learner engagement. In this sense, the study contributes both empirical evidence and a preliminary theoretical refinement of engagement as a construct framework in the context of GenAI-supported learning.

## 6 Limitations and future research

Several interpretive boundaries should be acknowledged when considering the findings of this qualitative multiple-case study. First, the data collection was preceded by a one-hour training seminar that introduced participants to ChatGPT’s functionalities and effective prompting strategies. This created a lightly scaffolded instructional context. Consequently, the observed engagement patterns may partly reflect this pedagogical framing, influencing participants’ expectations of what ChatGPT should do, their willingness to trust AI-generated feedback, and the specific uptake patterns later observed, rather than capturing purely unprompted or natural engagement.

Second, consistent with its exploratory qualitative design, the study did not include a baseline or comparison condition (e.g., an alternative feedback condition). Therefore, while the qualitative data provide rich, process-oriented insights into how learners interact with GenAI, the study cannot support definitive causal claims regarding anxiety reduction, writing improvement, or the relative pedagogical value of different feedback conditions.

Third, the findings are based on a small, context-specific sample of eight EAP learners from a single private institute in China. While appropriate for an in-depth case study, this limits the generalizability of the findings. Cultural, institutional, and disciplinary contexts may significantly shape how students perceive and engage with generative AI tools (Essien et al., 2024; Qu et al., 2024).

Fourth, although the study drew on multiple data sources—including screen recordings, interaction logs, and original and revised texts—parts of the analysis, particularly concerning cognitive and affective engagement, still relied on participants’ retrospective accounts during stimulated recall sessions and interviews. Despite their value in uncovering internal thought processes, these retrospective explanations may be subject to recall or social desirability biases and may not fully capture real-time reasoning.

To build on these findings, future research could observe learner engagement in less structured, unprompted contexts to better capture natural usage behaviours. It could also examine broader stages of the writing process, such as idea generation and drafting, or compare engagement patterns across different GenAI tools to develop a fuller understanding of AI-mediated learning.

## 7 Conclusion

This study examines the engagement patterns of eight EAP students in higher education as they interacted with ChatGPT during the revision of their L2 academic writing. By focusing on behavioural, cognitive, and affective engagement, the study provides process-oriented insights into how learners engaged with ChatGPT-mediated feedback in academic writing revisions. The findings suggest that ChatGPT-mediated feedback was associated with observable behavioural involvement and varied forms of cognitive engagement during revision. Participants queried feedback, revised their drafts, and, in some cases, iteratively refined their texts. Screen recordings and stimulated recall data further showed that some learners carefully evaluated suggested corrections and made reasoned decisions about whether and how to incorporate them into their writing. At the same time, these engagement patterns were uneven, with deliberate evaluation coexisting with instances of shallow uptake and dependence on the tool.

The affective dimension of engagement was more mixed. While many participants appreciated the immediacy and accessibility of ChatGPT’s feedback and described feelings of reassurance or confidence, others reported concerns about overreliance and felt that AI-mediated interaction offered less contextual and interpersonal support than teacher feedback. These findings point to the potential value of integrating ChatGPT as a complementary resource within teacher-mediated feedback practices, rather than treating it as a standalone substitute. Such an approach may allow learners to benefit from the immediacy and iterative affordances of GenAI-generated feedback while still receiving the pedagogical guidance and socio-emotional support associated with human instruction.

## Data Availability

The raw data supporting the conclusions of this article made available on request, without undue reservation.
