# Supplementary material for: Exploring English for academic purposes learners’ engagement with ChatGPT-4 in academic writing revision: a case study from a private language institute in China
Source: Front Artif Intell. 2026 Jun 1;9:1802007. doi: 10.3389/frai.2026.1802007 (PMC13267182; doi:10.3389/frai.2026.1802007)
Supplement: Supplementary file 1 [file Table_1.docx]

**Appendix A:** Various Stages of the Revision Process and SR (selected examples)

c

| **VideoTime** | **Original Text** | **ChatGPT’s comment** | **SR** |
| --- | --- | --- | --- |
| 4:29 | High pay means that I could provide a better life for my family – a good home, plenty of food, nice clothes, nice birthday gifts and money for my children’s education. | The word "nice" is a bit vague and informal. In an academic context, it’s better to choose more specific adjectives that provide clearer meaning.  Suggested revision (as already shown above): “thoughtful birthday gifts" instead of "nice birthday gifts.” | Accepted. But I am happy to illustrate more words. |
| 6:03 | A high salary would enable me to provide my family with a better quality of life, including a comfortable home, adequate food, good clothing, meaningful birthday gifts, and financial support for my children’s education. | Upgraded your sentense as follows: A well-paid job would allow me to provide a higher quality of life for my family, including a comfortable home, nutritious food, quality clothing, thoughtful birthday gifts, and adequate financial support for my children’s education. | Well paid jobs reads more naturally than high salary because I would inevitably make the same mistakes in my next writing session. …feels like having a native language expert mentoring me through the writing process, and I find it incredibly helpful and rewarding. |
| 8:18 | A well-paid job would allow me to provide a better quality of life for my family. With a stable and sufficient income, I could ensure that my family lives in a comfortable home and has access to nutritious food and quality clothing. Financial security would also allow me to celebrate important occasions, such as birthdays, with thoughtful gifts that show my appreciation and love.  More importantly, a higher income would enable me to invest in my children’s education. I would be able to provide them with better learning resources, extracurricular opportunities, and possibly support their future university studies. Education is one of the most valuable gifts parents can give their children, and having adequate financial resources would help me create more opportunities for their personal and academic growth. | Small Improvements for a Perfect Score (29–30)  Add more specific details or examples, such as:  • tutoring  • educational programs  • study abroad   - technology for learning   Example improvement:  For example, I could enroll my children in high-quality schools, provide them with books and educational  technology, and even  support them if they want to study abroad.  This makes the argument more concrete and  persuasive. | This revised version is excellent. It no longer has the tense errors I often make, and the logic flows much more smoothly now. I really like it. However, I ended up deleting one sentence at the end. |

**Appendix B**: Detailed uptake decisions by feedback category

| **Feedback category** | **Eileen A** | **Eileen D** | **James A** | **James D** | **Susan A** | **Susan D** | **Tanya A** | **Tanya D** | **Alice A** | **Alice D** | **Sara A** | **Sara D** | **Lawrence A** | **Lawrence D** | **Stafanie A** | **Stafanie D** |
| --- | --- | --- | --- | --- | --- | --- | --- | --- | --- | --- | --- | --- | --- | --- | --- | --- |
| **HOC: Style** | **25** | **0** | **20** | **6** | **19** | **2** | **17** | **1** | **11** | **0** | **12** | **0** | **9** | **1** | **2** | **1** |
| **HOC: Logic** | **0** | **0** | **0** | **0** | **1** | **0** | **3** | **0** | **0** | **4** | **0** | **1** | **0** | **0** | **0** | **2** |
| **LOC: Clarity / evidence / proofreading** | **0** | **9** | **0** | **3** | **10** | **2** | **19** | **8** | **10** | **2** | **1** | **1** | **2** | **3** | **12** | **4** |
| **LOC: Verb tense** | **2** | **1** | **1** | **2** | **0** | **3** | **0** | **0** | **8** | **2** | **10** | **0** | **1** | **3** | **9** | **3** |
| **LOC: Singular–plural** | **4** | **2** | **0** | **0** | **0** | **0** | **0** | **0** | **1** | **0** | **1** | **0** | **10** | **0** | **7** | **0** |
| **LOC: Spelling** | **0** | **2** | **0** | **1** | **2** | **0** | **8** | **0** | **0** | **0** | **0** | **0** | **7** | **1** | **2** | **0** |

**Appendix C**. Final coding framework for learner engagement with ChatGPT

| **Engagement dimension** | **Theme / category** | **Definition** | **Typical indicators** | **Illustrative example** |
| --- | --- | --- | --- | --- |
| Behavioural | Deliberate uptake | The learner selectively incorporates ChatGPT feedback after comparison or judgement | targeted revision, selective acceptance, modification before use | revising a vague lexical item after judging the suggestion more appropriate for academic writing |
| Behavioural | Mechanical uptake | The learner accepts feedback with limited evidence of evaluation | repeated acceptance, convenience-driven revision, rapid incorporation | accepting multiple revisions mainly to save time rather than to understand the change |
| Behavioural | Rejection of feedback | The learner does not implement ChatGPT feedback | refusal to revise, explicit non-use, retention of original wording | rejecting a punctuation suggestion because it conflicts with prior genre knowledge |
| Cognitive | Critical evaluation | The learner actively assesses the accuracy, appropriateness, or relevance of AI feedback | comparison with prior knowledge, genre-based reasoning, questioning AI output | evaluating a suggested change against model texts or prior EAP knowledge |
| Cognitive | Reflective uptake | The learner uses ChatGPT output as a stimulus for thinking, idea development, or interpretation | connecting AI output to personal experience, reflective elaboration, meaning-making | using an AI-generated example to develop an argument topic in relation to real-life experience |
| Cognitive | Tool-dependent processing | The learner reflects on AI output but relies heavily on it during idea generation or revision | dependence during planning, limited independent filtering, borrowing AI direction | depending on ChatGPT to generate initial examples before being able to start writing |
| Affective | Reassurance and confidence | The learner experiences emotional comfort, reduced anxiety, or increased confidence through ChatGPT use | relief, reassurance, feeling supported, lowered grammar anxiety | describing ChatGPT as a “virtual teacher companion” that reduces writing anxiety |
| Affective | Frustration and mismatch | The learner experiences negative emotions when AI output does not align with intentions or expectations | irritation, disappointment, concern over opacity, prompt–output mismatch | feeling frustrated when a brainstorming prompt produces a complete essay instead of ideas |
| Affective | Anxiety about dependence | The learner worries about overreliance on ChatGPT or loss of autonomy | concern about dependency, insecurity without tool access, fear of falling behind | expressing worry about not being able to write independently without paid access to ChatGPT |
| Affective | Preference for human support | The learner values teacher guidance more highly for context-sensitive or pedagogically sequenced support | desire for step-by-step explanation, interpersonal scaffolding, contextual trust | contrasting ChatGPT’s instant output with a teacher’s gradual and tailored guidance |
